# Estimation of cardiovascular risk and detection of subclinical carotid atheromatosis in patients with diabetes without a history of cardiovascular disease

**DOI:** 10.1590/2359-3997000000234

**Published:** 2017-01-27

**Authors:** Walter Masson, Salvador De Francesca, Micaela Molinero, Daniel Siniawski, Andrés Mulassi, Frank Espinoza Morales, Melina Huerin, Martín Lobo, Graciela Molinero

**Affiliations:** 1 Buenos Aires Argentina Council of Epidemiology and Cardiovascular Prevention “Dr. Mario Ciruzzi” of the Argentine Society of Cardiology, Buenos Aires, Argentina

**Keywords:** Diabetes, cardiovascular risk estimation, carotid atherosclerotic plaque

## Abstract

**Objectives:**

Cardiovascular risk estimated by several scores in patients with diabetes mellitus without a cardiovascular disease history and the association with carotid atherosclerotic plaque (CAP) were the aims of this study.

**Materials and methods:**

Cardiovascular risk was calculate using United Kingdom Prospective Diabetes Study (UKPDS) risk engine, Framingham risk score for cardiovascular (FSCV) and coronary disease (FSCD), and the new score (NS) proposed by the 2013 ACC/AHA Guideline on the Treatment of Blood Cholesterol. Ultrasound was used to assess CAP occurrence. A receiver operating characteristic (ROC) analysis was performed.

**Results:**

One hundred seventy patients (mean age 61.4 ± 11 years, 58.8% men) were included. Average FSCV, FSCD and NS values were 33.6% ± 21%, 20.6% ± 12% and 24.8% ± 18%, respectively. According to the UKPDS score, average risk of coronary disease and stroke were 22.1% ± 16% and 14.3% ± 19% respectively. Comparing the risks estimated by the different scores a significant correlation was found. The prevalence of CAP was 51%, in patients with the higher scores this prevalence was increased. ROC analysis showed a good discrimination power between subjects with or without CAP.

**Conclusion:**

The cardiovascular risk estimated was high but heterogenic. The prevalence of CAP increased according to the strata of risk. Understanding the relationship between CAP and scores could improve the risk estimation in subjects with diabetes.

## INTRODUCTION

The presence of type 2 diabetes mellitus approximately doubles the risk of cardiovascular mortality when compared with individuals without diabetes (1).

Previously, published studies showed that the cardiovascular prognosis of patients with diabetes without acute myocardial infarction (AMI) was similar to patients without diabetes but with a history of AMI (2-4). Consequently, the third National Cholesterol Education Program (NCEP) expert panel report on elevated blood cholesterol detection, assessment and treatment in adults (Adult Treatment Panel III – ATP III), considered the patient with diabetes as “coronary equivalent” (5). However, other reports have not confirmed these findings, generating controversy over what is the real cardiovascular risk of subjects with diabetes without coronary disease history (6-8).

A number of cardiovascular risk functions or scores have been developed from large epidemiological studies in general population (9-11). However, the low number of people with diabetes in the cohorts that originated these scores, puts limits its applicability. One of the few risk scores specifically developed in a population with diabetes came from the United Kingdom Prospective Diabetes Study (UKPDS risk engine) (12).

The estimation of cardiovascular risk in patients with diabetes would have clinical implications. Patients with diabetes, with or without additional risk factors or target organ damage, are considered “very high risk” or “high risk” respectively, with different LDL-C goal recommended in both groups, according to European guidelines for the management of cholesterol (13).

On the other hand, the 2013 American College of Cardiology/American Heart Association (ACC/AHA) Guideline on the Treatment of Blood Cholesterol recommends a new risk score (NS) in the population with diabetes without cardiovascular disease, suggesting moderate or high doses of statins according to the estimated risk (< or ≥ 7.5% respectively) (14).

There is evidence that the presence or absence of subclinical carotid atherosclerotic detected by ultrasound, improve the prediction of cardiovascular events in general population as well as in diabetes population (15-17).

Cardiovascular stratification with several risk scores, the association with the presence of carotid atherosclerotic plaque (CAP) and their implications on the use of statins have been previously evaluated by our working group in a primary prevention population in our country, but this analysis did not include patients with diabetes (18).

Therefore, the aims of the study were: 1) To stratify the cardiovascular risk using four different risk scores in patients with diabetes without cardiovascular disease history; 2) To estimate the correlation and concordance between these risk scores; 3) To describe the prevalence of CAP in the different risk categories according to each score; 4) To establish the optimal cutoff point (OCP) of each score that allows us to discriminate between subjects with or without CAP.

## MATERIALS AND METHODS

### Subjects

A multicenter, descriptive, cross-sectional study was performed on consecutive samples obtained in the cardiovascular prevention outpatient clinics of five cardiology centers in Buenos Aires, Argentina.

Subjects with diagnostic of diabetes were included in the study (fasting plasma glucose concentration ≥ 126 mg/dL in two consecutive measurements or plasma glucose ≥ 200 mg/dL on a 2-hour oral glucose tolerance test). Exclusion criteria were: 1) previous cardiovascular disease defined as AMI, prior percutaneous coronary intervention or coronary artery bypass graft surgery, or stroke or peripheral artery disease history; 2) chronic renal disease stage 4-5 (creatinine clearance < 30 mL/min); 3) concomitant lipid lowering therapy. The variables age, gender, total cholesterol, cholesterol bound to high-density lipoproteins (HDL-C), cholesterol bound to low-density lipoproteins (LDL-C), triglycerides, body mass index, systolic and diastolic blood pressure, duration of diabetes, hemoglobin glycosylate (HbA1c), family history of early coronary heart disease, smoking, presence of atrial fibrillation, and pharmacotherapy were collected.

### Cardiovascular scores and CAP

Four risk scores were calculated: 1) The Framingham 10-year risk score for cardiovascular disease (coronary death, AMI, coronary insufficiency, angina, ischemic stroke, hemorrhagic stroke, transient ischemic attack, peripheral artery disease, heart failure) based on lipids (FSCV) (19). 2) The Framingham 10-year risk score for coronary disease (FSCD) (9). 3) The NS for cardiovascular disease (AMI and stroke, fatal and nonfatal) used by the 2013 ACC/AHA Guideline on the Treatment of Blood Cholesterol (14). We consider indication of statins at moderate or high doses according to the estimated risk (< or ≥ 7.5% respectively). 4) The UKPDS 10-year risk score. The risk of coronary heart disease and stroke (fatal and nonfatal) was calculated. The UKPDS risk engine (ver. 2.0) was downloaded from the website and used to analyze the data (20).

The scores were analyzed by quartiles and the distribution was graphed using Kernel density estimation.

Ultrasound was used as noninvasive method for detecting the presence of CAP. The following points were required for the characterization of the plaque: 1) abnormal wall thickness (intima-media thickness > 1.5 mm), 2) abnormal structure (protrusion towards the lumen, loss of alignment with the adjacent wall) and 3) abnormal wall echogenicity. Carotid atherosclerotic plaque prevalence was compared between the different risks strata (quartiles) in the different scores used.

### Statistical analysis

A receiver operating characteristic (ROC) analysis was carried out to determine the area under the curve, assessing the four scores accuracy to discriminate between subjects with or without CAP. The Youden index [maximum vertical distance between the ROC curve and the line of statistical chance (CJ point)] was used to determine the score OCP. Continuous data were compared between groups using the t test for normal distribution or the Mann-Whitney-Wilcoxon test for non-normal distribution. The analysis of categorical data was performed using the chi-square test. Pearson’s test was used to obtain correlation between scores. The concordance between the FSCV, the FSCD, the NS and the UKPDS score for coronary disease was analyzed to classify patients into “high” or “non-high” risk strata (≥ 20% or < 20%), using the Fleiss kappa index. Mild or poor, acceptable or discrete, moderate, significant or almost perfect agreement was defined if the kappa value was < 0.20, between 0.21 and 0.40, 0.41 and 0.60, 0.61 and 0.80 and 0.81 and 1, respectively. Continuous variables were expressed as mean ± standard deviation, and categorical variables as percentages. A two-tailed p value < 0.01 was considered as statistically significant. STATA 11.1 and 3.1 EPIDAT software packages were used for statistical analysis.

### Ethics considerations

The study was conducted following the recommendations in medical research suggested by the Declaration of Helsinki, Guidelines for Good Clinical Practice and valid local ethical regulations.

## RESULTS

A total of 170 patients (mean age 61.4 ± 11 years, 58.8% men) were included in the study. Average body mass index was 30.6 ± 5 and mean total cholesterol, LDL-C, HDL-C and triglyceride values were 201 ± 36 mg/dL, 121 ± 34 mg/dL, 46 ± 13 mg/dL and 173 ± 101 mg/dL respectively. Mean HbA1c value was 7.0% (53 mmol/mol), 15% received insulin therapy and average duration of diabetes was 7.2 ± 6.5 years. Sixty four percent of patients were receiving antihypertensive treatment, 18.9% was active smokers and only 3.6% have history of atrial fibrillation. The baseline characteristics of the population stratified by sex are described in Table 1.

**Table 1:** Characteristics of the population stratified by sex

Continuous variables, mean (SD)	General population			Population with CAP		
	
	Women n = 70	Men n = 100	p	Women n = 26	Men n = 60	P
Age, years	61.5 (10.1)	61.0 (11.3)	0.76	68.1 (7.1)	66.8 (8.0)	0.47
BMI, kg/m^2^	31.6 (6.1)	29.9 (5.3)	0.04	31.5 (6.3)	29.4 (5.0)	0.11
Total cholesterol, mg/dL	197.8 (29.6)	202.7 (40.2)	0.36	202.5 (30.9)	202.3 (39.0)	0.98
LDL-C, mg/dL	113.9 (27.4)	126.3 (37.5)	0.01	118.3 (24.3)	129.8 (38.2)	0.1
HDL-C, mg/dL	51.3 (15.2)	41.8 (10.4)	< 0.001	48.6 (14.3)	42.3 (10.1)	0.05
Triglycerides, mg/dL	155.4 (73.3)	184.3 (115.7)	0.07	165.4 (71.3)	156.9 (67.9)	0.6
HbA1c, %	6.9 (1.4)	7.1 (1.2)	0.33	7.2 (1.4)	7.2 (0.9)	0.81
FSCV, %	20.6 (12.6)	42.4 (21.3)	< 0.001	28.3 (10.5)	51.7 (19.1)	< 0.001
FSCD, %	14.9 (7.2)	24.4 (13.4)	< 0.001	18.5 (6.0)	29.3 (13.2)	< 0.001
NS, %	16.2 (13.7)	30.6 (18.2)	< 0.001	25.3 (12.3)	39.4 (16.3)	< 0.001
UKPDS for CD, %	12.5 (8.6)	28.6 (17.1)	< 0.001	18.6 (9.6)	36.5 (16.7)	< 0.001
UKPDS for stroke, %	8.4 (8.4)	18.3 (22.4)	< 0.001	13.4 (9.8)	26.9 (25.3)	< 0.001
UKPDS for fatal CD, %	8.4 (7.5)	19.6 (15.4)	< 0.001	13.6 (8.8)	26.8 (15.4)	< 0.001
UKPDS for fatal stroke, %	1.3 (1.4)	2.7 (3.3)	< 0.001	1.9 (1.4)	3.9 (3.8)	< 0.001
Categorical variables, %						
Insulin therapy	10.1	18.2	0.15	7.7	21.7	0.14
Antihypertensive treatment	55.7	70.0	0.06	73.1	81.7	0.37
Smoking	10.0	25.0	0.001	15.4	30.0	0.03
Atrial fibrillation	0.0	3.1	0.08	0.0	8.3	0.32
Family history of early CD	10.0	23.0	0.03	7.7	21.7	0.12

SD: standard deviation; BMI: body mass index; FSCV: Framingham score for cardiovascular disease; FSCD: Framingham score for coronary disease; CD: coronary disease; NS: new score proposed by the 2013 ACC/AHA Guideline on the Treatment of Blood Cholesterol.

**Figure 1:**
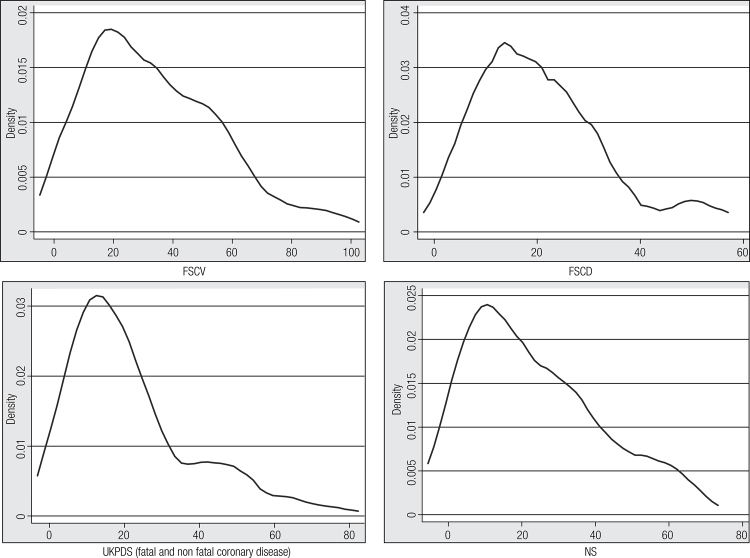
Kernel density distributions of the risk scores values.

FSCV: Framingham score for cardiovascular disease; FSCD: Framingham score for coronary disease; CD: coronary disease; NS: new score of the 2013 ACC/AHA Guideline; UKPDS: United Kingdom Prospective Diabetes Study.

Average FSCV, FSCD and NS values were 33.6% ± 21%, 20.6% ± 12% and 24.8% ± 18%, respectively. According to the UKPDS score, average risk of coronary disease (fatal and nonfatal), fatal coronary disease, stroke (fatal and non fatal) and fatal stroke were 22.1% ± 16%, 15.1% ± 14%, 14,3% ± 19%, and 2.1% ± 3%, respectively. The Kernel density distributions of the risk scores are showed in Figure 1.

A significant correlation was found between the estimations of all scores compared, with a range of “r” value between 0.46 and 0.98 (Table 2). However, the agreement (concordance) between the scores in categorizing the population as “high risk” or “no high risk” was moderate (kappa statistic between 0.45-0.59).

**Table 2:** Correlations between different risk scores values

	FSCV	FSCD	UKPDS (CD)	UKPDS (fatal CD)	UKPDS (Stroke)	UKPDS (fatal stroke)	NS
FSCV	–	r = 0.89	r = 0.87	r = 0.81	r = 0.58	r = 0.59	r = 0.84
FSCD	r = 0.89	–	r = 0.78	r = 0.71	r = 0.46	r = 0.48	r = 0.72
UKPDS (CD)	r = 0.87	r = 0.78	–	r = 0.98	r = 0.73	r = 0.72	r = 0.86
UKPDS (fatal CD)	r = 0.81	r = 0.71	r = 0.98	–	r = 0.78	r = 0.78	r = 0.86
UKPDS (stroke)	r = 0.58	r = 0.46	r = 0.73	r = 0.78	–	r = 0.98	r = 0.74
UKPDS (fatal stroke)	r = 0.59	r = 0.48	r = 0.72	r = 0.78	r = 0.98	–	r = 0.74
NS	r = 0.84	r = 0.72	r = 0.86	r = 0.86	r = 0.74	r = 0.74	--

FSCV: Framingham score for cardiovascular disease; FSCD: Framingham score for coronary disease; CD: coronary disease; NS: new score proposed by the 2013 ACC/AHA Guideline on the Treatment of Blood Cholesterol.

Subjects with CAP were older (67.2 ± 7.7 vs. 55.3 ± 10.0 years, p < 0.001) and evidenced higher prevalence of male sex (69.8% vs. 48.2%, p = 0.004), smoking (25.6% vs. 12.1%, p = 0.002) and anti-hypertensive treatment (79.1% vs. 49.4%, p < 0.001) than patients without CAP. The mean duration of diabetes was higher in subjects with CAP in comparison with patients without CAP (9.3 ± 7.6 vs. 4.9 ± 4.3 years, p < 0.001).

Mean scores values were significantly higher in patients with CAP (FSCV: 44.6% ± 20.0% vs. 22.1% ± 15.2%, p < 0.001; FSCD: 26.0% ± 12.5% vs. 14.9% ± 8.6%, p < 0.001; NS: 35.1% ± 16.5% vs. 13.9% ± 11.8%, p < 0.001; UKPDS for coronary disease: 31.1% ± 17.0% vs. 12.6% ± 7.9%, p < 0.001; UKPDS for fatal coronary events: 22.8% ± 15.0% vs. 6.9% ± 5.2%, p < 0.001; UKPDS for stroke: 22.8% ± 22.6% vs. 5.3% ± 4.8%, p < 0.001; UKPDS for fatal stroke: 3.3% ± 3.6% vs. 0.79% ± 0.98%, p < 0.001) compared with the group without CAP.

**Figure 2:**
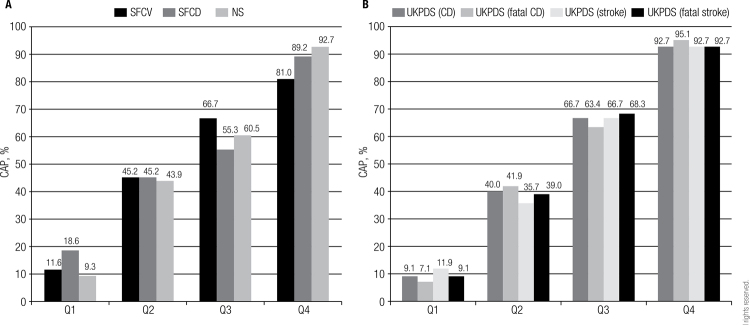
Prevalence of CAP according to different risk scores quartiles. (A) FSCV, FSCD and NS. (B) UKPDS scores.

CAP: carotid atherosclerotic plaque; FSCV: Framingham score for cardiovascular disease; FSCD: Framingham score for coronary disease; CD: coronary disease; NS: new score of the 2013 ACC/AHA Guideline; UKPDS: United Kingdom Prospective Diabetes Study; Q: Quartile.

Overall, the prevalence of CAP was 51% (men: 60%; women 38%), being greater in the higher risk strata (quartiles) in all the scores evaluated (Figure 2). Sixty three percent of diabetic patients with ≥ 7.5% of NS have CAP and only one subject with a NS < 7.5% have CAP.

Applying the NS, 80.4% of the population obtained a cardiovascular risk ≥ 7.5% (men 88.9% vs. women 68.1%, p = 0.001). Thus, considering the 2013 ACC/AHA Guideline on the Treatment of Blood Cholesterol, ≈ 80% of the population had absolute indication for high doses of statin therapy (98.9% in patients with CAP).

**Figure 3:**
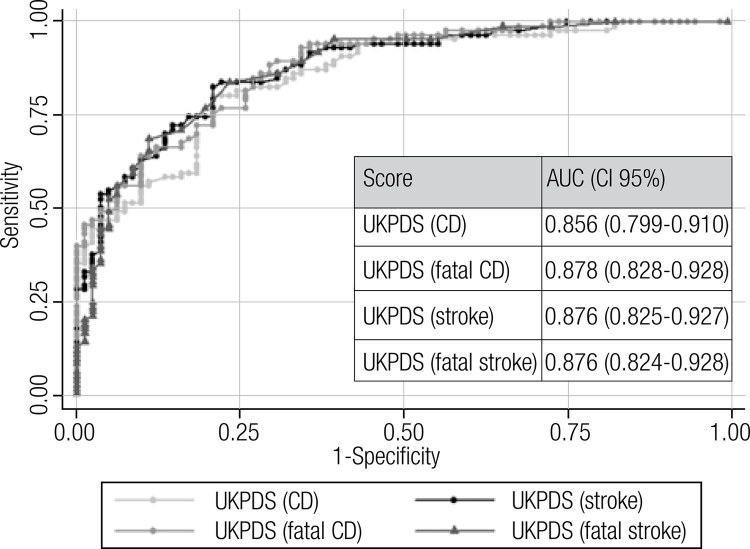
Discrimination capacity of the UKPDS scores between subjects with or without carotid atherosclerotic plaque (ROC analysis).

AUC: area under the curve; CI: interval confidence; CD: coronary disease; UKPDS: United Kingdom Prospective Diabetes Study.

**Figure 4:**
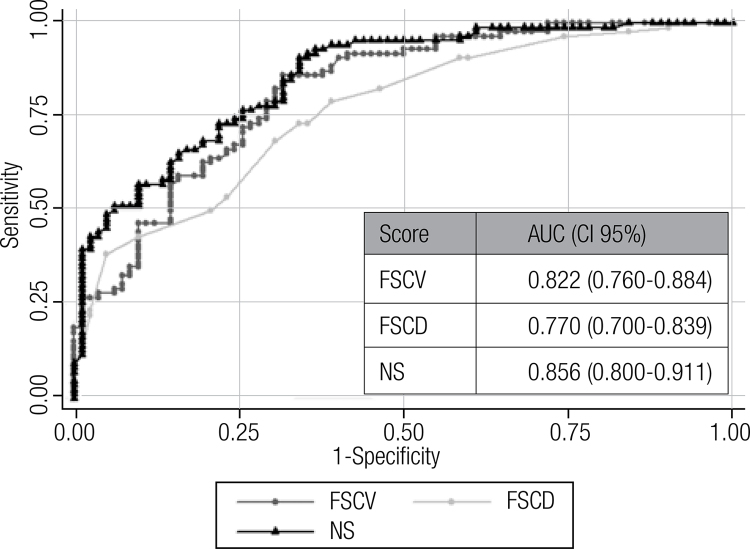
Discrimination capacity of the FSCV, FSCD and NS between subjects with or without carotid atherosclerotic plaque (ROC analysis).

AUC: area under the curve; CI: interval confidence; FSCV: Framingham score for cardiovascular disease; FSCD: Framingham score for coronary disease; NS: new score of the 2013 ACC/AHA Guideline.

ROC analysis showed a good discrimination power between subjects with or without CAP (Figures 3 and 4). When we analyze the population by sex, the area under the curve showed a good discrimination both in women (SFCV: 0.804, SFCD: 0.738, NS: 0.837, UKPDS score for coronary disease: 0.845, UKPDS for stroke: 0.839, UKPDS for fatal coronary disease: 0.858, UKPDS for fatal stroke: 0.820) and men (SFCV: 0.814, SFCD: 0.769, NS: 0.863, UKPDS score for coronary disease: 0.860, UKPDS for stroke: 0.900, UKPDS for fatal coronary disease: 0.885, UKPDS for fatal stroke: 0.915).

The OCP value of SFCV, SFCD and NS were 25.4% (sensitivity 86%, specificity 69%, Youden 0.547); 16% (sensitivity 79%, specificity 62%, Youden 0.405) and 14.3% (sensitivity 91%, specificity 66%, Youden 0.566) respectively. On the other hand, the OCP value of UKPDS score for coronary disease, fatal coronary disease, stroke and fatal stroke were 17.7% (sensitivity 80%, specificity 79%, Youden 0.592); 8,4% (sensitivity 90%, specificity 69%, Youden 0.587), 7.7% (sensitivity 83%, specificity 79%, Youden 0.616) and 1.1% (sensitivity 84, specificity 77%, Youden 0.603) respectively. When we analyze the population by sex, the OCP values were higher in men in comparison with women (SFCV: 31.0% vs. 15.9%; SFCD: 27% vs. 15.0%; NS: 22.4% vs. 11.8%; UKPDS score for coronary disease: 23.5% vs. 12.0%, UKPDS score for fatal coronary disease 13.1% vs. 7.1%; UKPDS score for stroke: 12.5% vs. 7.3% and UKPDS score for fatal stroke: 1.7% vs. 0.7%).

## DISCUSSION

The identification of patients at risk of developing cardiovascular events is one of the most challenging issues in clinical practice. Different risk assessment tools have been proposed to estimate the risk of future events. Although, the Framingham and UKPDS scores are the most frequently used. Recently, a new score has been proposed by the latest ACC/AHA Guideline on the Treatment of Blood Cholesterol. In our study, we compared these predictive scores in a group of patients with diabetes without a history of cardiovascular disease, including the NS, and then we analyzed its relationship with the presence of CAP.

According to what is seen in our results, the estimation of coronary risk at 10 years by using the FSCD and UKPDS score for coronary events is close to the theoretical 20% accepted as “coronary equivalent”. Moreover, when scores that predict combined cardiovascular events (FSCV or NS) are used, cardiovascular risk clearly exceeded this threshold. Additionally, stroke risk calculated by the UKPDS score was considerably (14.2%). Our findings were similar to the results of a study conducted in Spain. Using the UKPDS score, the authors found that the risk of coronary events and stroke was 23% and 12% respectively (21).

Although different endpoints were evaluated by each score, the Kernel density distribution on the FSCV, FSCD, NS and UKPDS for coronary events showed similar distributions. This finding is probably related with the good correlation found between different risks estimations. Similarly, a Spanish study showed a significant correlation between the REGICOR equation and UKPDS score in a group of subject with diabetes (22). However, in our study the agreement between the different scores was moderate. Diabetes patients were not classified into the same risk score categories. Therefore, we could not classified into the same categories all diabetes patients using different risk scores.

The prevalence of CAP in our study was 51%. This finding was consistent with previous reports. For example, Ahn and cols. reported a prevalence of 47% in subjects with diabetes in Korea (23) and Catalan and cols. showed a high CAP prevalence (60%) in new-onset diabetes subjects (24). Furthermore, in a group of patients with diabetes but with high cholesterol levels, the prevalence of CAP was even higher (69%) (25).

As expected, patients with CAP had worse clinical risk factors and higher values of all scores than subject without CAP. Our working group had already reported these findings in a cohort that did not include individuals with cardiovascular disease neither people with diabetes (18). Cardoso and cols. showed that in patients with diabetes, older age, male sex, smoking status and the results of ambulatory blood pressure monitoring were the main independent predictors of ultrasonographic carotid atherosclerosis (26).

In our study, the prevalence of CAP was higher in men than in women. This finding could be explained in part by the presence of a worse lipid profile and a higher prevalence of smoking and family history of coronary disease in men.

The prevalence of CAP was elevated in the highest quartiles of all the risk scores. Similarly, in the previously mentioned study, Hong and cols. reported that the prevalence and the number of carotid artery plaques were significantly higher in the high-risk group according to UKPDS risk stratification (25). However, we found that in the lowest quartile of all the scores the prevalence of CAP was between 9 to 19%. Then, a low score does not exclude the possibility of diagnose asymptomatic carotid atherosclerosis. This topic is relevant, especially in women, because the estimated cardiovascular risk for all scores was significantly lower than in men, even analyzing only the population with CAP.

In our investigation, all scores predict CAP with very good accuracy (area under the ROC curves above 0.75). Similarly, a study made in Japan showed area under the ROC curves of 0.76 and 0.79 for FSCV and UKPDS score respectively, for predicting coronary artery stenosis assessment with computed angiography (27).

Applying the NS, only one patient with a score value < 7.5% had CAP. Taking into account these findings, it seems that the recommendation proposed by the 2013 ACC/AHA Guideline on the Treatment of Blood Cholesterol regarding the indication of moderate or intensive doses of statins according to the risk level are appropriate. However, in our study the OCP of NS for the detection of CAP was 14.3%. This cutoff is about twice the one proposed by the NS to stratify patients with diabetes in the highest-risk category and to indicate intensive treatment with statins. This discrepancy might suggest that the cutoff point proposed by the NS in patients with DBT is too low and that the inclusion of the diagnosis of diabetes in the equation probably could increase excessively the impact of the disease on the estimation of risk of events. Something very similar happens in these guides, with the very strong dependence of ageing in the calculation of cardiovascular risk at 10 years. We consider that the equation used by the ACC/AHA Guideline does not capture the heterogeneity of risk present in diabetic patients and the detection of subclinical atherosclerosis could probably improve this limitation.

All other functions evaluated for coronary o cardiovascular events, the OCP was close or over 20%, threshold chosen to classify patients as high risk.

Finally, the OCP values were higher in men compared to women. These findings would suggest that the clinical applicability of the scores would be different in men and women, such as occurs in people without diabetes. However, the low number of individuals analyzed in each group, limiting the conclusions.

This study is associated with several limitations. First, it was cross-sectional with a small number of patients. Second, all participants were enrolled in cardiovascular prevention outpatient clinics of cardiology centers which may have introduced selection bias. Third, in our study, CAP was defined according to the Atherosclerosis Risk in Communities study criteria. Changing the definition of CAP could modify our results. Fourth, the low prevalence of atrial fibrillation in this population may have underestimation the risk of stroke. Finally, this study was not intended to determine whether risk classification was correct. A prospective study should be developed to confirm our findings.

Despite its limitations, our study represents a valuable contribution because we examined patients with diabetes from Argentina, whereas previous reports were limited to other regions of the world.

Several guidelines classify patients with diabetes as high or very high risk regardless of atheroma burden (5,13,28,29). Consequently, the evaluation of atherosclerotic burden by non-invasive imaging has not been definitely incorporated in clinical practice. However, some authors consider that carotid intima-media thickness is a useful marker of the progression of atherosclerosis and is an excellent predictor of cardiovascular events (30). Also, the Brazilian Diabetes Society recommended that patients with diabetes and without a history of cardiovascular disease should be stratified annually by the UKPDS risk-calculator (31). Additionally, through this tool, patients can be distributed between low risk (< 10% in 10 years), intermediate risk (10-20% in 10 years) and high risk (> 20% in 10 years). Furthermore, these recommendations suggest that coronary calcium score should be performed in patients with intermediate risk in order to reclassify their risk. In this context, the search CAP could be suitable for the same purpose, in situations that the computed tomography method is not available.

In conclusion, on average, the cardiovascular risk was elevated for all the scores that were evaluated. However, risk stratification was heterogenic. The prevalence of CAP increased significantly in the higher strata of estimated risk. Understanding the relationship between presence of CAP and scores could improve the estimation of risk in our patients with diabetes.
